# Into the metabolic wild: Unveiling hidden pathways of microbial metabolism

**DOI:** 10.1111/1751-7915.14548

**Published:** 2024-08-10

**Authors:** Özge Ata, Diethard Mattanovich

**Affiliations:** ^1^ Department of Biotechnology, Institute of Microbiology and Microbial Biotechnology BOKU University Vienna Austria; ^2^ Austrian Centre of Industrial Biotechnology Vienna Austria

## Abstract

Microbial metabolism has been deeply studied over decades and it is considered to be understood to a great extent. Annotated genome sequences of many microbial species have contributed a lot to generating biochemical knowledge on metabolism. However, researchers still discover novel pathways, unforeseen reactions or unexpected metabolites which contradict to the expected canon of biochemical reactions in living organisms. Here, we highlight a few examples of such non‐canonical pathways, how they were found, and what their importance in microbial biotechnology may be. The predictive power of metabolic modelling, well‐founded on biochemical knowledge and genomic information is discussed in the light of both discovery of yet unknown existing metabolic routes and the prediction of others, new to Nature.

## Graphical abstract

Despite the deep biochemical knowledge on microbial metabolism enhanced by genome sequencing, there is still a lot to discover in the uncharted depth of the metabolic wild. Discoveries are being made by chance, as well as by comprehensive (meta)genome mining and screening, and by metabolic model based pathway predictions.

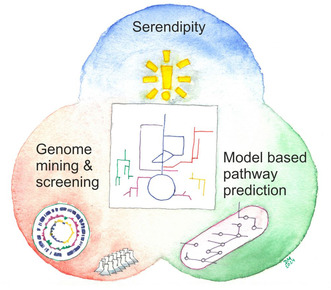

## FINDING THE UNSEEN: METABOLIC RESEARCH FAR FROM THE SHALLOW

Working in the times of fully sequenced genomes with functional annotations of their coding sequences, genome scale metabolic models, biological databases such as KEGG that link genome information with biochemistry and metabolism, suggests us that we have a near complete understanding of microbial metabolism. However, we witness surprises quite frequently, where new functions, new pathway routes, novel biochemical conversions, unexpected substrates or metabolic products are discovered. Obviously, there is a gap between our expectations towards our current understanding of microbial metabolism, and the reality of limitations due to underrated complexities of metabolic networks.

But what are the reasons for this gap between expectation and reality? One is certainly based in the often overlooked promiscuity of enzymes. Researchers have tended to annotate only one known function (substrate‐to‐product conversion) to enzymes. However, it is not uncommon that enzymes accept also other, similar metabolites as substrates, converting them to products that may not be part of the canonical metabolic networks. Another reason can be found in compartmentation—the same enzyme may run a reaction in opposite directions, for example, in different compartments. In this context, we should be aware that compartmentation is not fully annotated to date. Not all compartment target signals are clearly identifiable, and proteins may localize to several compartments, so that we must take into account that likely more pathways run at different equilibria in vivo than typically annotated.

### Why is that important for biotechnology?

The phenomenon of non‐canonical pathways is an ambivalent domain. In times of synthetic biology, it is a nuisance as we want to assume full predictability of metabolic reactions in a cell, and the responses to interventions by pathway engineering. In an ideal setup, metabolic models should be able to predict all metabolic fluxes in networks. However, experience tells that engineered pathways, additional substrates, or changes in external conditions can cause errors and uncertainties due to unknown reactions (or the unexpected lack of some). Finding unexpected reactions or pathways, on the other hand, can be an important enabler for metabolic engineering. It is not uncommon that such un‐annotated pathways were found serendipitously during pathway engineering and proved to be enablers for efficient production of biomolecules that were new to the host.

**FIGURE 1:**
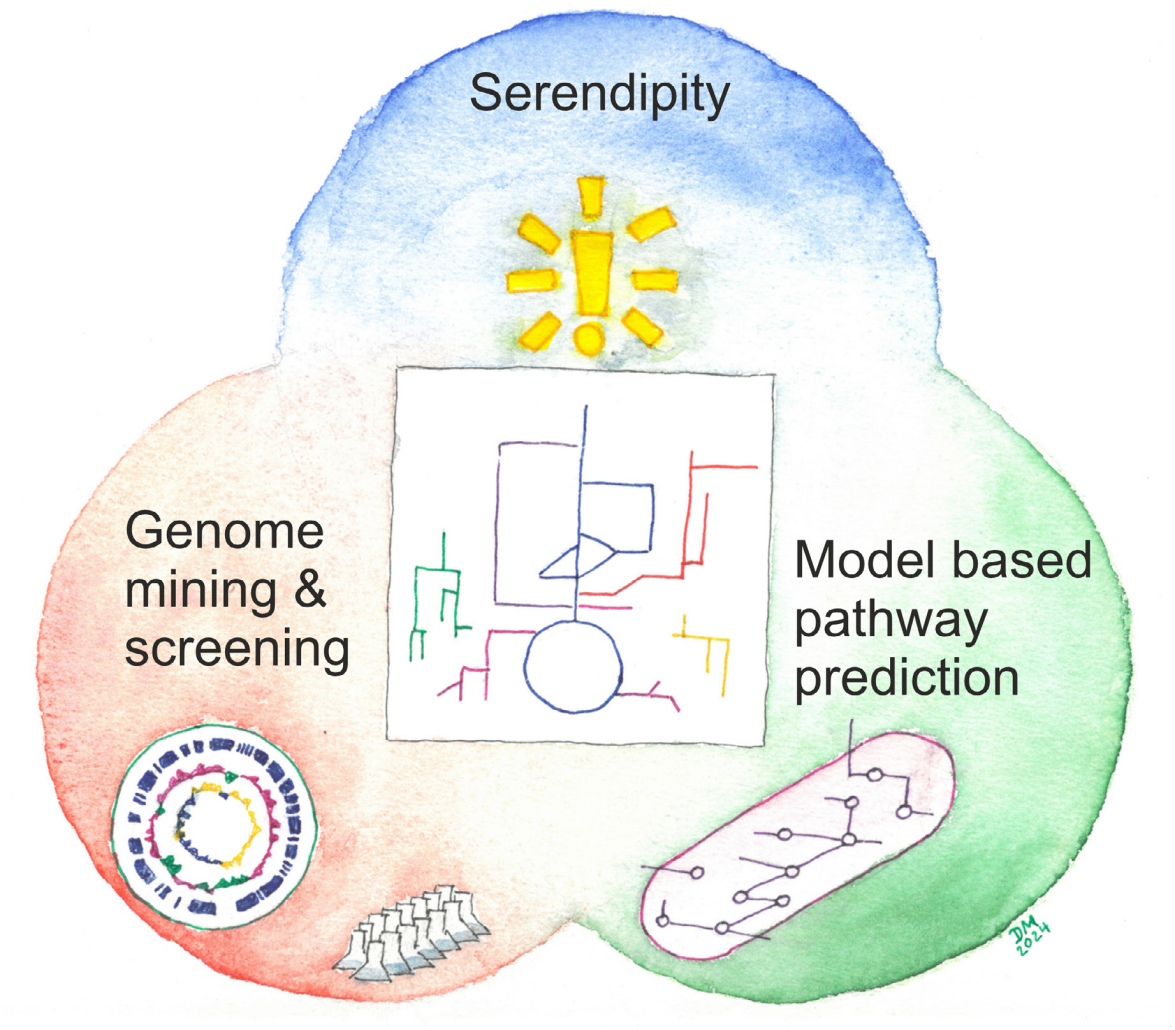
Identification of non‐canonical metabolic pathways. Most frequently, unexpected metabolic routes were found by chance—or serendipitously, in the sense of lucky coincidences, when experiments did not lead to an expected outcome. This process depends on chance, and on the readiness of researchers to realize an opportunity when it occurs. The genomic era has opened a route to search for novel enzymatic reactions and their potential links in pathways, typically combined with strain screening in conditions that are selective for investigated (groups of) reactions. Genome mining and screening still mostly search the known space, while computational metabolic modelling offers predictive power to design new pathways that might have not evolved in nature yet.

In the following, we highlight several examples of such unexpected, previously un‐annotated reactions and pathways, their identification, and potential benefits for metabolic engineering. These examples should be seen illustrative rather than a comprehensive review, and they illustrate how finding novel pathways is transitioning from lucky coincidences over strain screening to the predictive power of computational metabolic models (Figure 1).

### Central carbon metabolism

Microbial glucose metabolism has been deeply studied, and it is mainly attributed to two pathways, the Embden‐Meyerhof‐Parnas (EMP) and the Entner‐Doudoroff (ED) pathway, with additional bypasses through the pentose phosphate pathway (PPP). Their difference is mainly found in the yield of ATP and NADPH, respectively, where a higher NADPH (vs. NADH) output correlates with a lower ATP yield and a higher metabolic driving force. A further pathway, the periplasmic gluconate shunt (PGS) yields ATP but no reducing equivalents, and extracellular gluconate or 2‐ketogluconate that can be metabolized at a later timepoint. Although the pathway topologies have been well known, there is scarce information about the relative importance and regulation of these different pathways. With a smart combination of metabolic modelling and a two‐tracer study using ^2^D and ^13^C labelling, the spatial and temporal distributions of glucose flux was resolved in *Pseudomonas putida*, providing valuable information on the correlation of pathway distribution in relation to growth rate, and the potential benefits for environmental bacteria in their competitive habitats (Volke et al., 2023).

### Single carbon pathways

Microbial methanol utilization has been known since decades. The challenge to build each carbon–carbon bond de novo is met both in bacteria and some yeasts by several different, mostly cyclic pathways. The methanol assimilation pathway of yeasts has been described already in the 1970s to be based on xylulose 5‐phosphate (Xu5P) as the acceptor of the single carbon intermediate formaldehyde (Van Dijken et al., 1978). Xu5P is recycled by PPP reactions; however, it was for long not known how the recycling proceeds in detail. The key to identifying the pathway was a multi‐omics study of *Komagataella phaffii* which revealed that sedoheptulose 1,7‐bisphosphatase, a PPP enzyme in *Saccharomyces cerevisiae*, was highly induced on methanol, both at the transcriptional and the protein level. Further investigation revealed that all pathway reactions are catalysed by a set of peroxisomal enzymes, encoded partly by duplicated genes (Rußmayer et al., 2015). It is anticipated that the compartmentation enables the xylulose monophosphate cycle to run at different equilibria than the PPP, enabling performance at thermodynamically feasible concentrations.

Most single carbon assimilation pathways are cyclic. The co‐assimilation of a methyl group with CO_2_ by reversion of the glycine cleavage system, however, would be linear. It allows for assimilation of both methanol and formate, and it was initially designed as a de novo synthetic carbon fixation pathway (Cotton et al., 2018). It was soon after postulated to exist in nature by a metagenome study of wastewater treatment sludge (Figueroa et al., 2018), and further identified to really exist and function in *Desulfuvibrio desulfuricans* (Sánchez‐Andrea et al., 2020). Although these bacterial pathways depend on oxygen‐sensitive enzymes, an oxygen tolerant version of the reductive glycine pathway (rGly) could be demonstrated in yeasts (Bysani et al., 2024; Mitic et al., 2023). In *K. phaffii*, both methanol and formate are co‐assimilated with CO_2_ via the rGly pathway, and the deletion of just one competing reaction enabled growth on both, methanol and formate (Mitic et al., 2023). By the overexpression of *K. phaffii* pathway genes Guo et al. (2024) enabled growth in *S. cerevisiae* as well.

Tolerance to potentially toxic substrates is especially important for the assimilation of single carbon substrates. As systems level study of C1 metabolism in *P. putida* revealed several oxidoreductases that can oxidize methanol, formaldehyde and formate (Turlin et al., 2023). These toxic substances are no canonical substrates for *P. putida* but they can occur as by‐products of metabolism, and knowledge on their detoxification is a valuable information for engineering C1‐pathways into this organism.

Engineering a functional Calvin‐Benson‐Bassham cycle into heterotrophic microorganisms is seen as a landmark towards CO_2_ based bioproduction (Gassler et al., 2020; Gleizer et al., 2019). It has been unclear, however, how these synthetic autotrophs deal with phosphoglycolate, the product of the oxygenase side reactions of RuBisCO, a problem for which native autotrophs had millions of years of evolution to deal with. Only recently it was found by chance that the autotrophic *K. phaffii* strain uses promiscuous reactions of two enzymes, a lactate oxidoreductase and a phosphatase, to channel phosphoglycolate via glyoxylate into the central metabolism (Baumschabl et al., 2024).

### Metabolic product formation

The canonical lysine synthesis pathway has been described in the 1950s–1960s to proceed from aspartate semialdehyde and pyruvate as precursors to d,l‐diaminopimelate in six steps, whereby the amino group of tetrahydrodipicolinate is protected after ring opening during reduction and transamination either by succinate (in *Escherichia coli* and many Gram‐positive bacteria) or by acetate (in a few *Bacillus* species) (Peterkofsky & Gilvarg, 1961). Isotopic (^14^C) tracer analysis, however, revealed that *Corynebacterium glutamicum* performs a pathway that converts the intermediate piperideine‐2,6‐dicarboxylate directly to d,l‐diaminopimelate, without the chance of rotation of the symmetric intermediate l,l‐diaminopimelate (Leadlay, 1979). As l,l‐diaminopimelate was identified as a precursor of lysine in this species (Nakayama et al., 1966; Nakayama & Kinoshita, 1961) it was speculated that channelling of the pathway through a multi‐enzyme complex was responsible for the apparent ‘single pot’ reaction, however it turned out that a previously predicted (Oshima et al., 1964) single dehydrogenase reaction bypasses the canonical four reactions in *C. glutamicum* and some other bacteria (Ishino et al., 1984; Misono et al., 1979). The inconsistency of data was later clarified by the unexpected fact that *C. glutamicum* operates both the succinylase and the dehydrogenase pathways (Schrumpf et al., 1991). Besides the curiosity of the unexpected duplication of a metabolic pathway this is obviously relevant as it allows *C. glutamicum* to regulate its lysine synthesis more flexibly across growth phases and media, allowing for a simple single step bypass when conditions favour that. The succinylase pathway has further been exploited for carbon flux coupling of lysine production by making the strain addicted to diaminopimelate production for the TCA cycle to proceed (Kind et al., 2013).

Metabolic pathway design for production of biomolecules relies often on our knowledge of biochemical reactions and the enzymes catalysing them. We may, however, base design also on the conversion of non‐canonical substrates by enzymatic cascades to develop new, hitherto not described pathways. One example illustrating this strategy described the de novo design of a pathway to produce 2,4‐dihydroxybutyrate (DHB) with *E. coli*, using the enzymatic cascade from aspartate to homoserine as a blueprint to convert malate to DHB. With a combination of overexpression and engineering of both homologous and heterologous enzymes, efficient production of DHB as a non‐natural precursor for methionine could be demonstrated (Walther et al., 2017).

### Nitrogen and sulfur metabolism

Although the central carbon metabolism of many microorganisms is well understood, there is a considerable lack of knowledge about the assimilation of non‐canonical sources for other elements such as nitrogen, sulfur, or phosphorous. With a combination of parallelized strain screening and genome mining, the assimilation of choline (Linder, 2014), nitrate (Linder, 2019a), cyanate (Linder, 2019b), sulfamate (Linder, 2017), or aromatic organosulfur compounds (Linder, 2016) was clarified in different yeast species.

Bacteria have been described to perform 14 different redox reactions on 8 inorganic nitrogen species of different redox states, playing key roles in the assimilation and recycling of nitrogen (Kuypers et al., 2018). Many of the involved microbes are not cultivated yet and their study depends fully on analyses of environmental samples. Metagenome mining has become crucial for finding novel enzymes and pathways of microbial nitrogen cycles. By cloning and analysing large DNA fragments from soil and marine water samples it was demonstrated that very abundant Crenarchaeota contribute to nitrogen cycles with nitrite reductases and ammonia monooxygenases, previously not annotated to them (Hallam et al., 2006; Treusch et al., 2005). The metabolic potential of *Candidatus* Hydrothermarchaeota, an abundant marine microbe, has been explored by metagenome mining, highlighting its role in the cycling of carbon, nitrogen, sulfur, and iron in marine environments (Kato et al., 2019).

*P. putida* is known to assimilate various inorganic and organic nitrogen sources. As a free‐living soil bacterium, it depends on the flexibility to utilize a variety of nitrogen molecules depending on their respective availability. Schmidt et al. (2022) have combined genome mining by barcoded transposon sequencing with screening on drop‐out media for the ability to grow on 52 nitrogen sources, and identified 672 genes that are involved in the assimilation of inorganic and organic nitrogen sources.

These examples on nitrogen assimilation illustrate the power of (meta)genome mining, often combined with screening on minimal media to find new nitrogen substrates and to identify pathways to utilize them. They highlight both the power of metagenome analysis to find pathways in uncultured microorganisms and the value of high throughput methods such as pooled mutant fitness analysis to identify yet unknown pathways in a single, presumably well‐studied organism such as *P. putida*.

## HOW CAN WE HARNESS THE POTENTIAL OF NON‐CANONICAL PATHWAYS?

We build our knowledge about cellular metabolism on a huge collection of biochemical data gathered over decades of research. The full potential of all these data is leveraged by community‐based efforts, linking the data in genome scale databases, such as KEGG, BRENDA, or UniProt, through the combination of existing metabolic building blocks from various organisms. Besides such general databases, species specific resources such as RegulonDB for *E. coli* or SGD for *S. cerevisiae* provide additional specific insight on model organisms. Although the use of these databases relies on trusting them in principle, we need to be aware of their limitations: unresolved loopholes and errors in models, unexpected metabolic by‐products, or infeasible constraints can indicate a non‐canonical reaction or pathway in the metabolic network that may only become visible in an engineered strain under certain culture conditions. It is essential to keep the mind open for the unexpected, allowing for a critical reassessment and amendment or modification of current knowledge.

For example, an unknown bypass reaction was discovered in *E. coli* that catalyses sedoheptulose 7‐phosphate to sedoheptulose 1,7‐bisphosphate and consequently to dihydroxyacetone phosphate and erythrose 4‐phosphate in the PPP pathway, and verified by ^13^C labelling metabolic flux analysis (Nakahigashi et al., 2009). In another example, a so‐far hypothesized yet not‐proven reaction in PPP by a transketolase‐like protein 1 (TKTL1) was verified to occur in CHO cells based on parallel ^13^C MFA and integrated flux fitting to estimate the TKTL1 flux. These examples show the potential of using metabolic models as they show the ‘unexpected’ even in well‐studied model organisms (Ahn et al., 2016).

One major concern regarding genome data is the potential spread of false or inaccurate functional annotations iterating by sequence similarity‐based gene annotations where functions are ascribed by analogy to other organisms' genes rather than being experimentally verified. As the source of information on a putative gene function is not always clearly indicated in databases, we advise researchers to always trace the original literature related to a gene function to verify its plausibility in a given non‐model organism.

A special case is the genes with no known functional annotation in model organisms, like the y‐genes in *E. coli* (so named because their gene names begin with the letter y). Ghatak et al. (2019) used multiple databases to define the y‐ome of *E. coli* as the 35% genes that lack experimental evidence of their function, and highlight the differences between genes that lack any known function and those with attributed localization or computationally annotated functional domains. A similar situation is observed with eukaryotic genomes, where a consistent fraction of about 20% of the genes remains with unknown (or unstudied) gene products. Wood et al. (2019) compared unknown gene products of *Schizosaccharomyces pombe*, *S. cerevisiae*, and *Homo sapiens*, and concluded that these genes remained understudied even since genome sequences were published. They disprove the common assumption that unknown gene products are mostly orphaned, as the majority are conserved from yeasts to humans, and they conclude that the reasons for the lack of research on these proteins is two‐fold. For many of these genes, no apparent knockout phenotype was observed in standard conditions. On the other hand, researchers still focus on a few well‐studied proteins rather than exploring unknown territories, which the authors partly ascribe to risk‐adverse strategies of funders and reviewers.

Although the primary use of metabolic models is the analysis of already known metabolic pathways, the potential of metabolic models extends far beyond these analyses. They can be used to uncover hidden pathways, unknown reactions, and to construct de novo pathways. D'Ari and Casadesús (1998) have coined the term ‘underground metabolism’ for ‘reactions catalyzed by normal (unmutated) enzymes acting on substrate analogues which are themselves endogenous metabolites’, in other words, promiscuous reactions of enzymes within the native metabolic network. The underground metabolic network model of *E. coli* was reconstructed compiling 262 new reactions and 277 new compounds (Notebaart et al., 2014), and further extended in the light of metabolic engineering, adding nearly 300 more underground reactions (Kovács et al., 2022).

By utilizing the available databases, genome scale models and tools, a metabolic network is created that serves as a space with a set of compounds and enzymes enabling designing a de novo pathway tailored to a specific metabolite or product of interest. A review of available tools to design de novo pathways shows how a design‐build‐test‐learn approach can aid in achieving higher yields with novel pathways (Sveshnikova et al., 2022; Vayena et al., 2022). As a result, via leveraging existing data on enzyme kinetics, genes, and thermodynamic properties, it is possible to design an efficient metabolic network not (yet) discovered by Nature. Recently, an erythrulose monophosphate (EuMP) cycle for formaldehyde assimilation was designed, using a promiscuous dihydroxyacetone phosphate dependent aldolase as key enzyme (Wu et al., 2023). ^13^C MFA enabled to understand the differences of two evolved clones from a synthetic EuMP strain and revealed that these clones can find different metabolic solutions towards faster growth. This shows the diversity of metabolic adaptations of cells and offers great potential for metabolic engineering purposes.

Design of such synthetic pathways can also be an inspiration to search for them in Nature. In a search for a pathway for CO_2_‐fixation in *D. desulfuricans* (Sánchez‐Andrea et al., 2020), the researchers were able to find a seventh natural CO_2_‐fixing pathway based on a synthetic reductive glycine pathway that was proposed several years ago (Bar‐Even et al., 2013). These synthetic pathways can offer significant advantages over natural ones, such as reduced energy requirements or improved kinetic rates. An example for such a pathway is designed by Bar‐Even et al. (2010) in which two CO_2_ molecules are fixed to produce one molecule of glyoxylate via a shortcut version of the reductive TCA cycle with only four enzymes (Bar‐Even et al., 2010). However, even though such a pathway is stoichiometrically possible, it is thermodynamically infeasible. Even when synthetic pathways are thermodynamically possible, their implementation in vivo presents challenges. The robustness of newly constructed models must be thoroughly evaluated, considering factors such as stability, localization, regulation, and the specific process conditions under which the host organism will be cultivated. One example to this is the CETCH cycle which is a synthetic CO_2_ fixing pathway that comprises 17 enzymes from different domains of life with a reduced ATP requirement per CO_2_ (Schwander et al., 2016). However, so far, no in vivo implementation of the CETCH cycle could be realized.

## AN OUTLOOK TO THE FORESEEABLE FUTURE

Biochemical research has provided a solid foundation for our understanding of metabolic pathways. The advent of genome sequencing and annotation has substantially accelerated the mapping of these pathways, allowing us to uncover intricate details at an unprecedented pace. Moreover, metabolic modelling has emerged as an invaluable tool, enabling us to outline and predict the vast metabolic terrains with increasing accuracy. So, will we soon have explored the entire metabolic world? This seems unlikely, as the majority of microbial species are still uncultivated and many are yet to be discovered. To some disappointment also, the unknown fraction of cellular metabolism of model organisms has not been enlightened as much as anticipated. These metabolic depths are matched by underground metabolism by unannotated reactions of known enzymes. A clever combination of analytical tools such as isotopic tracer analysis with computational pathway mapping and modelling, however, will further accelerate the quest to yet uncharted depths of microbial metabolism.

## AUTHOR CONTRIBUTIONS

**Özge Ata:** Conceptualization; funding acquisition; writing – original draft; writing – review and editing. **Diethard Mattanovich:** Conceptualization; funding acquisition; writing – original draft; visualization; writing – review and editing.

## CONFLICT OF INTEREST STATEMENT

The authors declare no competing interests.

## Data Availability

Data sharing is not applicable to this article as no new data were created or analyzed in this study.
